# *Stenosternus* Karsch, a possible link between Neotropical and Afrotropical Orphninae (Coleoptera, Scarabaeidae)

**DOI:** 10.3897/zookeys.335.5471

**Published:** 2013-09-24

**Authors:** Andrey Frolov

**Affiliations:** 1 Zoological Institute, Russian Academy of Sciences, Sankt-Petersburg 199034, Russia

**Keywords:** Trans-Atlantic dispersal, Africa–America disjunction, biogeography, scarab beetles, orphnines, Orphnini, Aegidiini, São Tomé, Gulf of Guinea, Cameroon line

## Abstract

The monotypical orphnine genus *Stenosternus* Karsch is known from a single specimen of *Stenosternus costatus* collected on the São Tomé island (Gulf of Guinea). The holotype of *Stenosternus costatus* Karsch is re-examined and its characters are discussed and illustrated. Although the genus was implicitly placed by Paulian (1984) in the Old World tribe Orphnini Erichson, re-examination of the holotype of *Stenosternus costatus* shows that it has characters similar to those of the members of the New World tribe Aegidiini Paulian. Placement of *Stenosternus* in the Aegidiini is supported by the metepisternum widened posteriorly (forming posterior metepisternal lock for closed elytra) and a keel separating basal and anterolateral parts of propleurae. Relationships of *Stenosternus* with other orphnine taxa and possible ways of origin of São Toméan orphnine fauna are discussed.

## Introduction

Orphninae Erichson is a group of predominantly tropical scarab beetles. To date, 15 genera and 195 species of Orphninae are known (Frolov 2012) and they are classed into two tribes, Aegidiini Paulian comprising four Neotropical genera and Orphnini Erichson comprising the rest of the genera distributed in the Old World (Paulian 1984). The subfamily includes the monotypical genus *Stenosternus* Karsch which was originally described as the “Copridae” (Karsch 1881) but later placed in the “Orphnidae” (Karsch 1887). According to Paulian’s (1984) classification, the genus should be considered in the tribe Orphnini because it originates from the Old World. However Paulian did not discuss characters of the genus and how they fit diagnoses of the tribes and apparently he never saw the type specimen of *Stenosternus costatus* Karsch, 1881. In his earlier work Paulian (1948) wrote that *Stenosternus* was unknown to him.

*Stenosternus costatus*,the only described species of the genus,has a puzzling combination of unique characters with those similar to the members of the Aegidiini. The original description of the genus and another work clarifying its position (Karsch 1887) do not include many characters of potential phylogenetic importance. Except for peculiar middle and hind tarsi, no characters were illustrated and the genus has not been compared with other genera of the Orphninae except for preliminary phylogenetic analysis which showed possible relationships with Neotropical taxa rather than Afrotropical (Frolov 2012). However, at that stage it was not possible to examine the taxon in more detail. The present work is aimed at filling this gap. Below the redescription of the holotype is given and its characters are discussed and illustrated. Relationships of *Stenosternus* with other orphnine taxa and possible ways of origin of the São Toméan orphnine fauna are also discussed.

## Material and methods

Material used in this work is deposited in or borrowed from the following organizations: Institut royal des Sciences naturelles de Belgique, Bruxelles (IRSNB), Muséum d’Histoire Naturelle, Geneva (MHNG), Museum für Naturkunde, Humboldt-Universität, Berlin (MHUB), Muséum national d’Histoire naturelle, Paris (MNHN), Natural History Museum, London (NHML), Zoological Institute RAS, Saint-Petersburg (ZIN).

Preparation of genitalia follows the common technique used in entomological research. Photographs were taken with a Leica MZ9.5 stereo microscope and a Leica DFC290 digital camera from dry specimens. Partially focused serial images were combined in Helicon Focus software (Helicon Soft Ltd.) to produce completely focused images. Photographs were not altered except for digital enhancing with Adobe Photoshop (Adobe Inc.): levels and tone correction, background elimination. Outline figures were made by tracing features on digital photographs with Adobe Illustrator (Adobe Inc.).

For modeling the distribution of *Aegidium* Westwood, MaxEnt software (Elith et al. 2011) was used with the following settings: maximum number of background points – 10000, replicates – 1, replicated run type – crossvalidate, output format – logistic. The distribution model was based on 31 localities of *Aegidium* specimens derived from the literature (Cartwright and Chalumeau 1978; Morón 1991; Paulian 1984) and from the labels of the collection specimens. Country or province records without more precise localities and doubtful data were not used. Two sets of environmental layers were used: 18 bioclimatic variables (Hijmans et al. 2005; available from http://www.worldclim.org) and 17 soil property variables derived from ISRIC World Soil Information database (available from http://www.isric.org). Preparation of environmental layers and visualization of the Maxent distribution model was done with ArcGIS software (ESRI Inc.).

## Redesription

### Genus *Stenosternus* Karsch, 1881.

**Type species.** *Stenosternus costatus* Karsch, 1881, by monotypy.

#### Stenosternus costatus

Karsch, 1881

http://species-id.net/wiki/Stenosternus_costatus

##### Male, holotype

**Figures 1–10.**
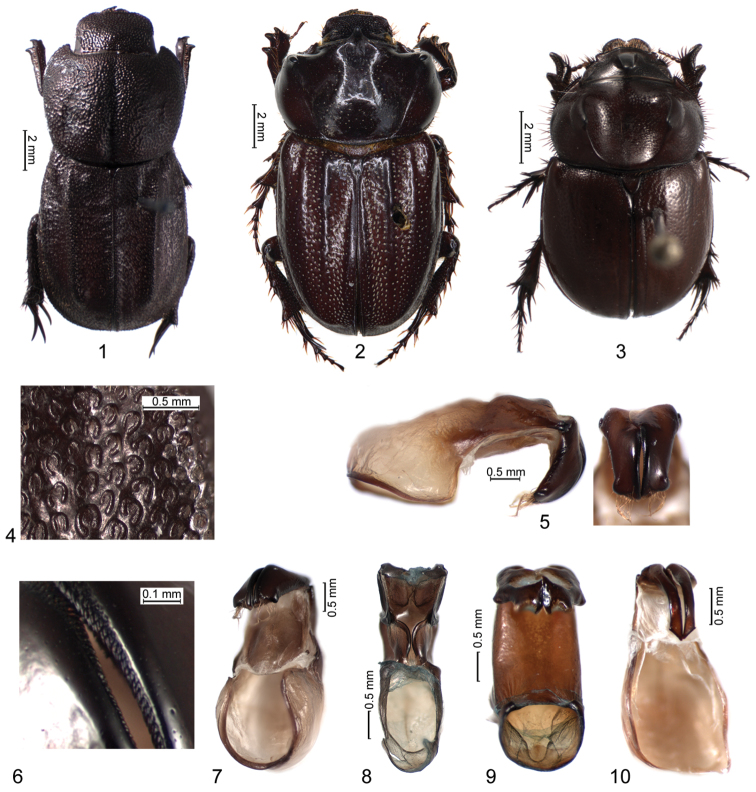
**1–3** Habitus of males **4** punctation of elytral disc **5** aedeagus in lateral view and parameres in dorsal view **6** serration of medial margins of parameres **7–10** aedeagus in ventral view **1, 4–7** *Stenosternus costatus* Karsch, holotype **2** *Aegidium colombianum* Westwood **3** *Orphnus macleayi* Laporte de Castelnau **8** *Hybalus cornifrons* (Brullé) **9** *Aegidium parvulum* Westwood **10** *Orphnus compactus* Petrovitz.

(MHUB). Body length (from anterior margin of clypeus to apices of elytra) 16.1 mm, pronotal width 7.3 mm, elytral width 7.6 mm. Body (Fig. 1) uniformly dark-brown with slight bronze tint. Surface densely punctate, almost rugose. Head and most part of pronotum punctate with oval deep punctures separated by 0.5 to 0.2 their diameters, sometimes almost adjacent. Each puncture has shagreened microsculpture with 1 short (only slightly protruding above surface of pronotum) seta. Intervals between punctures look smooth.

Clypeus emarginate anteriorly, with crenate margin, without tubercles. Genal and frontoclypeal sutures absent. Genae not protruding past eyes, indistinct. Frons feebly convex medially. Labrum feebly protruding past clypeus. Eyes relatively small, eye width 1.5 times smaller than distance between eye margin and gula (in ventral view).

Antenna 10-segmented, with 3-segmented club.

Pronotum trapezoidal, elongated (1.25 times wider than long) while in other Orphninae it is normally wider and shorter (about 1.6 times wider than long, Fig. 2, 3). Pronotum with distinct longitudinal middle depression from base to almost anterior margin. Lateral margin crenulate, base not bordered. Punctation of pronotum is similar to that of head. Propleurae with fine carinae separating anterolateral areas from basal area adjacent to bases of elytra (Fig. 34, arrowed).

Anterior tibiae relatively slender, almost parallel-sided, with 2 short lateral teeth and a smaller medial tooth (Fig. 15). Anterior tarsi absent. Anterior coxa with relatively deep longitudinal fossa on ventral side. Middle and hind legs similar in shape. Middle and hind femora relatively slender, almost parallel-sided, punctate with elongate punctures. Middle and hind tibiae without ridges on outer sides, rugosely punctate. Middle tibiae have 2 apical spurs, outer spur about twice as long as inner one. Hind tibiae with 2 spurs (outer spur about 1.5 times longer than inner one) and with modified spur-like basal tarsomere (Fig. 13). Tarsi of middle tibiae are absent but according to the picture provided by Karsch (1887) the specimen had middle tarsi modified to spurs similar to those of hind legs. Stridulatory area on hind coxae present.

**Figures 11—37.**
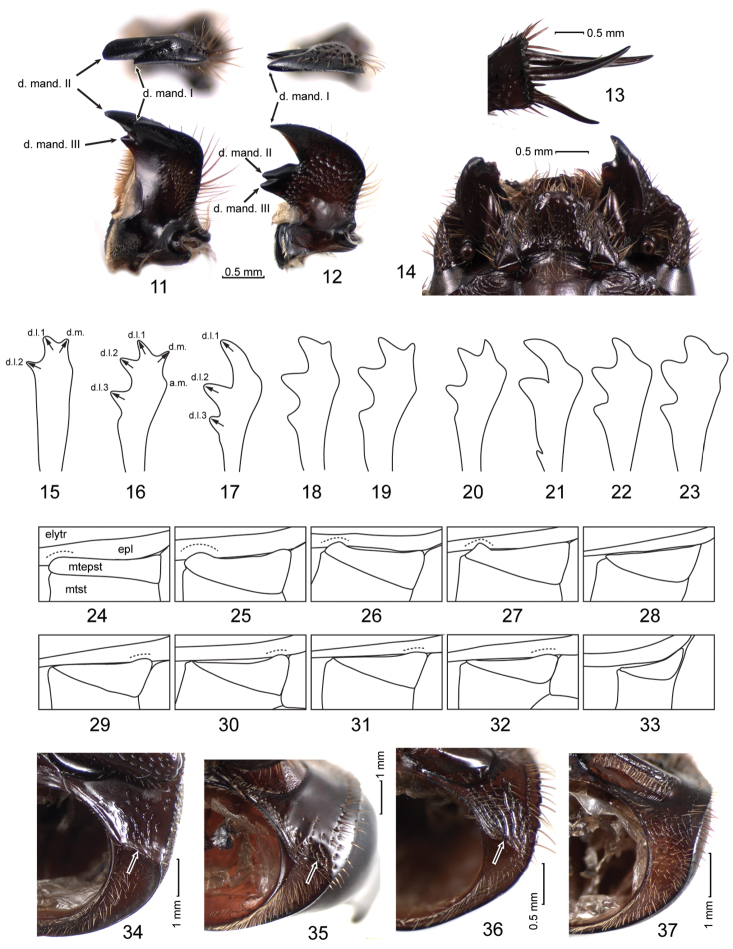
**11, 12** Right mandible in dorsal and apical view **13** apex of metafemur **14** head in ventral view **15–23** protibia of males (arrows in figures **15–17** indicate approximate teeth direction) **24–33** metepisternum **34–37** base of prothorax (arrows in figures **34–36** indicate keel separating basal and anterolateral parts of propleurae) **11, 16, 35** *Aegidium colombianum* Westwood **12, 37** *Orphnus declivis* Schmidt **13–15, 24, 34** *Stenosternus costatus* Karsch, holotype **17, 29** *Orphnus macleayi* Laporte de Castelnau **18, 36** *Aegidinus guianensis* (Westwood) **19, 27** *Aegidiellus alatus* (Laporte de Castelnau) **20, 26** *Paraegidium costalimai* Vulcano et al. **21, 31** *Pseudorphnus hiboni* Paulian **22, 30** *Triodontus itremoi* Paulian **23** *Chaetonyx robustus* Schaum **25** *Aegidium parvulum* Westwood **28** *Orphnus giganteus* Paulian **32** *Renorphnus clementi* (Petrovitz) **33** *Hybalus cornifrons* (Brullé).

Elytra somewhat oblong, 1.2 times longer than width. Humeral umbones small but distinct. Elytra without striae but each elytron with low longitudinal ridge from base to about 5/6 its length. Elytra densely punctate with characteristic semicircular punctures each bearing a short setae (Fig. 4). Because of rugose punctation, lateral margin of elytra appear crenulate in dorsal view. Epipleuron with concavity receiving hind margin of metepisternum.

Wings vestigial, about 1/2 length of elytra.

Scutellum 1/20 length of elytra, narrow, rounded apically.

Metepisternum narrow, almost parallel-sided, with rounded distal part which somewhat overlaps epipleuron (Fig. 24). Orifice between mesocoxal cavities absent. Abdominal sternites with irregularly shaped punctures, some punctures V-shaped. Abdominal sternite 8 longer than others, without concavity or tubercle in the middle. Plectrum trapezoidal, with minute seta in the middle near apical margin.

Aedeagus with heavily sclerotized parameres about 2 times shorter than phallobase (Fig. 5, 7). Phallobase symmetrical, with sclerotized ventral plate separated by weakly sclerotized membranous areas (Fig. 7). Apices of parameres with tooth-like rounded processes directed anterolaterally and relatively long and dense pale setae. Lateral sides of parameres with short, sparse, mostly abraded setae. Medial sides of each paramere without membrane in apical 2/3 but with dense, scale-like teeth directed apically (Fig. 6). Basal 1/3 of inner margin of parameres overlapping. Endophallus with about 15 small, tooth-like spinules, without larger sclerites.

##### Diagnosis.

*Stenosternus* can be easily distinguished from other Orphninae by the combination of uniquely modified legs and sculpture of pronotum and elytra. However, this diagnosis is based on a single male specimen and may include characters of sexual dimorphism. Aedeagus with serrated medial sides of parameres, setose apices of parameres, and separate ventral sclerite of phallobase is also highly distinctive.

## Discussion

### Morphological traits of *Stenosternus*

**Mouthparts**

Paulian (1984) separated Aegidiini from Orphnini largely on the basis of their distribution and the shape of the mouthparts, especially the mandibles. Aegidiini were described as having simplified mandibles with reduced teeth on the inner side as opposed to having well developed inner teeth in the Orphnini. However, mandibles of American taxa cannot be considered simplified in comparison to the Old World taxa. For example, right mandibles of *Aegidium colombianum* Westwood (Fig. 11) and *Orphnus declivis* (Fig. 12) both have 3 inner teeth but the shape of the incisor part is different: in *Aegidium* the teeth are somewhat clustered distally and the mandibular tooth II is the largest and actually apical while in *Orphnus* incisor part is larger and the tooth I is apical. Mandibles of *Aegidiellus alatus* (Laporte de Castelnau), *Paraegidium costalimai* Vulcano et al. and *Aegidinus guianensis* (Westwood) are similar to those of *Aegidium*, although *Aegidinus* has large lateral processes. In the Orphnini, the diversity of mandible shapes is greater which probably reflects a wider spectrum of food types utilized by the beetles.

In the type specimen of *Stenosternus costatus*, right mandibular apex is worn and probably broken (Fig. 14) but the mandible shape is more similar to that of *Aegidium* than of *Orphnus*. It can be seen that the bristled incisor comb occupies a large part of the inner margin of the mandible.

Although the mandible morphology is probably species-specific in some orphnine genera, mandibular characters should be attributed low weight in reconstructing phylogeny of the groups above genus level. The morphology of the mandibles and other mouthparts depends heavily on the type of food utilized by the species. Adaptive radiation might result in parallel modification of the mouthparts in different lineages. Using mandibles in phylogenetic reconstructions is further complicated by their manifold variability not normally found in other structures: interspecific variability, asymmetry, sexual dimorphism, allometric variability in males (i.e. *Madecorphnus* Paulian), and variability caused by wearing during adult individual life. It is also important that mouthparts be studied as an interdependent complex of structures with strong morpho-functional relationships with each other. Adaptation to a particular food type results in regular and interrelated modifications in all mouthparts including labrum. Undesired redundancy can be introduced in a dataset if these characters are coded separately and, if not weighted, they can overcontribute to the most parsimonious tree topology.

**Legs**

The fore legs of *Stenosternus costatus* lack tarsi. The absence of protarsi is often found in Coleoptera including Scarabaeoidea; sometimes only males lack protarsi. However, in the Orphninae well developed protarsi are present in all groups except for *Stenosternus*. The shape of the *Stenosternus* protibiae is also unusual for the Orphninae: they are narrow, almost parallel-sided, with three short teeth (Fig. 15). These teeth can be interpreted as the three common scarabeoid lateral teeth with the tooth I being offset medially. However, in all Old World taxa these teeth are directed laterad of the imaginable midline of a protibia (Fig. 17, 21–23; approximate teeth direction are arrowed). In the males of the New World genera (except possibly for *Paraegidium*, Fig. 20) there is an additional, mostly shorter, medial tooth directed mediad of the protibia midline (Fig. 16, 18, 19). A similar medial tooth can be found in *Chaetonyx robustus* (Fig. 23), although it is less distinct and apparently subject to a reasonable interspecific variability. Although such medial teeth in male protibiae are known in other scarabeoid groups (i. e. in scarabeine genera *Macroderes* Westwood, *Xinidium* Harold and *Metacatharsius* Paulian), within the Orphninae they may be homologous.

One of the most prominent characters of *Stenosternus* is the modification of middle and hind tarsi which are reduced to only basal tarsomeres similar to spurs in length and shape (Fig. 13). Tarsal origin of these “spurs” can be seen from their setation and was discussed by Karsch (1887). In scarab beetles, spurs can be pectinate apically but never bear setae on their sides while tarsomeres normally do. Such modification is unique not only among orphnines but also among other scarab beetles. The middle and hind legs of *Stenosternus*, including the apical spurs, are otherwise of normal shape, symmetrical, and do not look malformed.

The shape of the legs of *Stenosternus costatus* suggests that the beetles are poorly adapted to digging in soil. Slender tibiae with short lateral teeth (in protibiae) and without transverse keels (in meso- and metatibiae) are typical for Passalidae and Lucanidae which have apparently ancestral habit as rotten wood dwellers. No information is available about biology of *Stenosternus costatus* but morphology of the specimen suggests that it too may be a rotten wood dweller. This also agrees with available data about *Aegidium cribratum* Bates which, at least in Mexico, is found only in rotten logs of a few species of tree (Morón 1991), whereas no Afrotropical, Madagascar, or Palearctic species were recorded from rotten wood.

The adaptive significance of the absence of tarsi is not always clear. It can be speculated that the tarsi are lost when there is no need for them, for example when a beetle does not clamber onto plants. Apparently a large number of Orphninae species, especially flightless ones, do not leave upper soil layer and litter and have no need in clambering. However all taxa, except for *Stenosternus*, have well developed tarsi in all legs. The absence of tarsi in *Stenosternus* might be an adaptation for defense from predators like ants or soldier termites.

**Pronotum**

The pronotum of *Stenosternus* is distinctive in being longer than in other Orphninae. Its width is almost equal to length (and it looks longitudinal in dorsal view) while in other taxa it is 1.5 times or more wider than long. It lacks ridges or tubercles which are found in males of most Orphninae taxa. A shallow but distinct longitudinal middle depression is found only in *Stenosternus*. The pleural areas of the pronotum have a feature which is not found in other Old World Orphninae. The propleura have fine but distinct carinae separating anterolateral areas from basal area adjacent to bases of elytra. This area is similar to that in all American genera which have basal area of pronotum adjacent to elytra separated from the rest of propleuron (Fig. 35, 36). Old World taxa have the propleura smoothly convex without any signs of carinae (Fig. 37). The shape of the pronotum of *Stenosternus* and American genera apparently allows for tighter joining of the pronotum and mesonotum which might be an antipredator adaptation.

**Integument and punctation**

*Stenosternus costatus* has a characteristic sculpture of the dorsal body surface. Its head and pronotum are densely and rugosely punctate with relatively large, mostly adjacent punctures. In other Orphninae, the punctation of the head and especially pronotum is normally finer and sparser. More distinctive is the punctation of the elytra which are densely covered by U-shaped punctures directed caudally (Fig. 4). Orphninae show reasonable variability of elytral punctation but in most taxa punctures are more or less round. Somewhat similar U-shaped punctures are known in *Paraegidium* but they are directed apically and their homology is unlikely. The elytra of *Stenosternus costatus* also have characteristic longitudinal ridges. Similar ridges are found in *Aegidium* but in this genus each elytron has 2 ridges (Fig. 2). Other taxa do not feature such elytral ridges.

**Brachyptery**

The type specimen of *Stenosternus costatus* has vestigial wings about 1/2 the length of elytron. Flightlessness is quite common in the Orphninae and the total number of flightless species may be up to 20 per cent (Frolov, unpubl.). *Hybalus* Brullé and *Chaetonyx* Schaum comprise completely apterous species with no visible wing rudiments whereas a number of *Orphnus* MacLeay species have vestigial wings similar to those of *Stenosternus*. Flightless species are also known among *Aegidium* (i.e. *Aegidium parvulus* Westwood from Guadeloupe).

**Metepisternum**

The metepisternum shows reasonable variability among orphnine genera (Fig. 24–33). In most taxa it is more or less triangular and tapering caudally (in this paper only the part of metepisternum not covered by elytron is discussed and illustrated) (Fig. 28–33). In most of these groups the metepisternum has a slightly widened anterodorsal angle slightly overlapping epypleuron which is somewhat concave in this place (Fig. 29–32). This structure, anterior metepisternal lock, apparently serves to hold the closed elytra more securely. It is absent in *Hybalus* (Fig. 33) which might be a secondary loss due to aptery, but it is also indistinct in *Orphnus giganteus* Paulian (Fig. 28) which has fully developed wings. In the New World taxa, the anterior metepisternal lock is less developed and sometimes indistinct but there is a similar structure in posterior angle of metepisternum which is rounded to triangular and is situated in the distinct concavity of epypleuron (Fig. 25–27). Such a posterior metepisternal lock is not found in the Old World genera except for *Stenosternus*. The metepisternum of *Stenosternus* is dissimilar to that in other orphnines in being narrow, almost parallel-sided in most part, but its posterior angle is rounded and overlaps slightly the epypleuron (Fig. 24). A shallow but distinct concavity can be seen on the epypleuron. It is possible that the posterior metepisternal lock is reduced in *Stenosternus costatus* due to brachyptery and it was more developed in its flying ancestors. In the preliminary phylogenetic analysis of the Orphninae (Frolov 2012) the posterior metepisternal lock was interpreted as a synapomorphy of *Stenosternus* and American genera.

**Aedeagus**

The aedeagus of *Stenosternus costatus* has a few distinctive features. Scale-like serration on medial sides of each paramere in apical 2/3 is unique at least among Orphninae. The phallobase of *Stenosternus costatus* can be classed as one of the four types of phallobase found in the Orphninae. It has a ventral sclerite about 2 times shorter than dorsal sclerite. The two sclerites are separated ventrolaterally by thinner, feebly sclerotized membranes (Fig. 7). This type of phallobase is similar to that of *Hybalus* where ventral sclerite is also distinct, but in *Hybalus* it has a complex shape (Fig. 8). In the four American genera, the phallobase is tube-shaped with strongly sclerotized ventral side but without differentiation of ventral and dorsal sclerites (Fig. 9). The majority of other Orphninae genera have a phallobase of the forth type which is strongly sclerotized dorsally and with a thin membrane ventrally (Fig. 10). Apices of parameres of *Stenosternus costatus* are densely setose. Similar setation can be found also in the monotypical American genus *Aegidiellus*, and Mediterranean genus *Hybalus*. Apparently in the Orphninae, setation of parameres is genus-specific, but its homology and phylogenetic value is not clear due to high variability of paramere shape which is, in general, species-specific.

### Distribution and possible habitat of *Stenosternus costatus*

The only known specimen of *Stenosternus costatus* was collected by Richard Greeff on the São Tomé island in the Gulf of Guinea. São Tomé is the largest island of the oceanic sector of the Cameroon Line of volcano-capped swells. It lies 240 km offshore and has never been connected to mainland Africa. The age of São Tomé is estimated as 13 MY (Lee et al. 1994) although it is a minimum estimate based on the age of the oldest exposed volcanic rocks. São Tomé, Príncipe, and Annobon are classed as one of the WWF 200 ecoregions with exceptional richness of biodiversity. The original vegetation of São Tomé comprises forests of various types, including lowland and montane forests. São Tomé along with the other islands of Gulf of Guinea is considered a rain forest refuge since the mid-Miocene.

Precise collecting locality of the holotype of *Stenosternus costatus* is unknown. Krauss (1890) mentioned one locality in the island where Greeff had collected insects: Roça Rio d’Ouro. This is a plantation (roça) in the northern part of the island at elevation of about 180 m, surrounded by a forest. It is possible that *Stenosternus costatus* was collected in this locality or elsewhere in the north-eastern foothills of the escarpment.

### Biogeography of the Orphninae and possible origin of São Tomé orphnine fauna

The six recent regional faunas of the Orphninae (Frolov 2012) are separated by different dispersal barriers. Of them, fauna of São Tomé is the smallest one comprising only one species of the genus *Stenosternus*. However it is quite distinctive because the genus is endemic to the island and has putative relations to the New World taxa.

The core fauna of the islands of the Gulf of Guinea is of African origin. Bioko (Fernando Po) is the largest island with the most diverse biota though it is of continental origin and was connected to mainland Africa during the last glaciation maximum. Consequently its fauna differs slightly from the fauna of the mainland Cross-Sanaga-Bioko coastal forests. Of the true oceanic islands, São Tomé is the largest (850 km^2^), separated from mainland Africa by 250 km, has a few climatic regions, complex terrain profile, and supports rich biota. Príncipe, although much older than São Tomé (originated in early Oligocene) has six times smaller area and two times smaller altitude range. Annobon is a small island (17 km^2^) lying 340 km off African shore and having relatively poor biota. Because the islands were apparently a target for colonization for a long time (since the Miocene in case of São Tomé), adaptive radiation resulted in high endemicity at specific and generic levels. The islands contain some of the highest percentage of endemicity in the world (Jones 1994).

Atlantic currents might facilitate dispersal of African biota to the oceanic islands of Gulf of Guinea (Renner 2004). John Measey et al. (2007) showed that favorable oceanic currents with reduction in salinity of surface waters might facilitate rafting from the Congo and Niger basins to the islands. Dispersal with rafts seems the only sound hypothesis to explain occurrence on São Tomé of such poor oceanic dispersers as shrews, as well as a high proportion of subterranean taxa amongst the herpetofaunal endemics (Drewes 2002; John Measey et al. 2007).

Of the all Gulf of Guinea islands, Orphninae have so far been recorded only from São Tomé. It is possible that some *Orphnus* species (e.g. *Orphnus gilleti* Benderitter) known from the neighboring mainland region will be found in Bioko. Annobon is unlikely to have any Orphninae due to its small size and apparently unsuitable habitats. However few studies are available on the insects of the Gulf of Guinea and scarab beetles of the islands were not surveyed.

Available data do not allow to soundly hypothesize about the way of separation of the New World and Old world Orphninae faunas. Of the all barriers between regional orphnine faunas, the Atlantic barrier was apparently the least permeable for the members of the group. South America–Africa disjunctions are commonly explained by either vicariance or long distance dispersal. Vicariance of the previously Gondwanan group due to the continent breakup seems the least probable hypothesis in the case of the orphnines. Distribution of the extant taxa shows no pattern of Gondwanan groups: they are absent from regions where Gondwanan relicts are commonly found including Notogea (Australasia) and Patagonian Province of Neotropical Realm. Afrotropical region is the contemporary center of diversity and possible center of origin and diversification of the group. In Africa, Orphninae are mainly distributed in the central part of the continent in savannah and forest–savannah mosaic regions. Few species were recorded south of 25°S.

It is possible that orphnines dispersed from Western Africa to north-eastern South America via the Atlantic ocean. Zoogeographical relationships of Gulf of Guinea islands with South America have been discussed in a few works and modern disjunctive ranges of a number of plant (Renner 2004) and animal (Carranza and Arnold 2006) taxa are explained by long distance dispersal. The islands of the Gulf of Guinea lie on this putative migration path since the early Miocene. Migrants might have ecize on the islands and *Stenosternus* might be the only survived descendant of these migrants. Putative habit of *Stenosternus* and its ancestors as rotten wood dwellers might facilitate dispersal over the ocean inside wood logs. Such dispersal seems less probable in case of geophilous taxa like *Hybalus* and *Chaetonyx*, as well as litter and upper soil layer dwellers to which most of Afrotropical, Madagascar, and South-East Asian taxa apparently belong.

**Figure 38.**
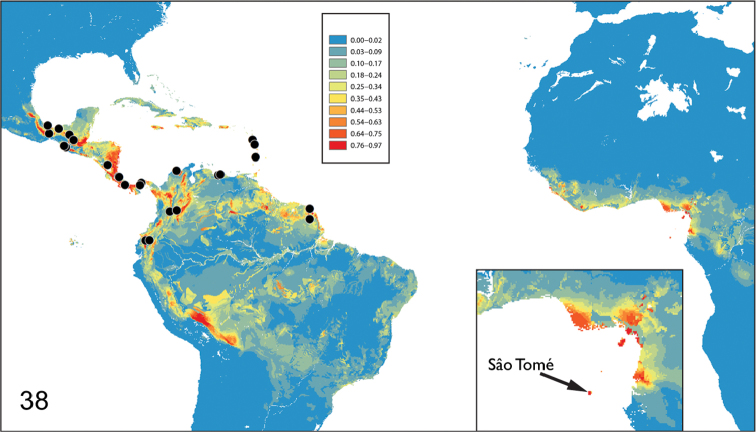
Representation of the MaxEnt distribution model for *Aegidium* Westwood. Warmer colors show areas with better predicted conditions. Black dots indicate known collecting localities of *Aegidium* used for the model. Insert shows the Gulf of Guinea and the São Tomé island. Other explanations are in text.

To analyze distribution pattern of the Orphninae, MaxEnt distribution models were created for a number of genera including *Aegidium* (Fig. 38). It is beyond the scope of the present work to analyze *Aegidium* model in detail but one aspect of it is relevant here: the model predicts high probability of environmentally suitable biotopes in the islands of Cameroon line including São Tomé. The model is based on current climatic conditions but despite reasonable fluctuation of climate in the Northern Hemisphere, especially in the Pleistocene, and fluctuation of the size of equatorial rain forests in Africa and South America, climatic conditions are thought to be relatively stable in the Gulf of Guinea and the islands are considered a rain forest refuge since the Miocene. The distribution model suggests that *Aegidium* and *Stenosternus* share similar type of habitat. This can be an additional argument for considering the two genera phylogenetically related rather than superficially similar.

### Taxonomic position of *Stenosternus* and phylogeny of the Orphninae

The data available to date do not allow *Stenosternus* to be definitely classifiedas a member of either Orphnini or Aegidiini. Paulian (1984) assumed it was a member of the Orphnini but he did not discuss it. Colby (2009) apparently followed Paulian. I provisionally moved *Stenosternus* to the tribe Aegidiini (Frolov 2012). The placement in the Aegidiini seems better justified as it is supported by a few putative synapomorphies: metepisternum widened posteriorly (forming posterior metepisternal lock for closed elytra) and a keel separating basal and anterolateral parts of propleura. The shape of the mandibles of *Stenosternus* is more similar to that of New World genera as is the fore tibia with a medial tooth. The two later characters however need confirmation on a larger material which is currently not available. In general appearance, *Stenosternus* resembles *Aegidium* which was also noticed by Kolbe (cited by Karsch 1887). On the other hand, *Stenosternus* lacks a hole connecting middle coxal cavities. This character was interpreted as a synapomorphy of the New World orphnine genera (tribe Aegidiini *sensu* Paulian: Frolov 2012) but the presence of the hole in *Aegidinus* requires confirmation because my observation was based on a single specimen and might be a preparation artifact. The phallobase of *Stenosternus* is not similar to that of the New World genera which all have rather uniform tube-shaped phallobase.

Phylogenetic relationships of *Stenosternus* require further research because the material currently available is limited to a single specimen. The study of a female would reveal sexual dimorphism of this taxon. Especially important would be the study of premature stages because the larvae of the New World taxa, at least the genus *Aegidium*, differ significantly from those of the Old World taxa (Barbero and Palestrini 1993; Morón 1991; Paulian and Lumaret 1982; Randriamanantsoa et al. 2010).

## Supplementary Material

XML Treatment for Stenosternus costatus
